# Stapleless Laparoscopic Sleeve Gastrectomy: a Relatively New Approach in Bariatric Surgery

**DOI:** 10.1007/s11695-016-2376-9

**Published:** 2016-09-27

**Authors:** C. Rajkumar Vinayak, Sivaneswaran Lechmiannandan, Umasangar Ramasamy

**Affiliations:** Department of Surgery, Taiping Hospital, Taiping, Perak Malaysia

Laparoscopic sleeve gastrectomy (LSG) has been established as an effective procedure for the treatment of morbid obesity gaining popularity over the last decade as an effective single procedure of bariatric surgery [1]. The cost incurred however has been of major concern as the stapler power gun and staple materials are expensive. Hence, the need arose for stapleless bariatric surgery. Jacques Himpens of Belgium pioneered stapleless bariatric surgery by performing a laparoscopic Roux-en-Y gastric bypass (LRYGB) utilizing the LigaSure Atlas (LSA) and over sewing the wound edges with non-absorbable synthetic sutures. He used this stapleless technique for 10 patients as laparoscopic procedures, such as duodenal switch, sleeve gastrectomy, and Roux-en-Y gastric bypass. The operating time was not significantly lengthened, and no ill effects were recorded [2].

In 2005 at the International Federation for the Surgery of Obesity and Metabolic Disorders (IFSO) meeting held in Maastricht, Netherlands, Almino Ramos and colleagues presented a video demonstration of a stapleless Roux-en-Y Gastric Bypass (RYGB) utilizing the LSA and went on to publish their work in 2006 [3]. In 2013, Masoud Rezvani and colleagues from Abington Memorial Hospital, Pennsylvania, published a single report on Robotic Stapleless LSG, and in February 2016 at the Annual Conference of Obesity and Metabolic Surgery Society of India (OSSICON) in India, Shashank Shah and colleagues presented a video of stapleless LSG [4]. Upon literature search, only the above-mentioned publications and presentations have described stapleless bariatric surgery. However, LSG technical details were not described in depth by any of the authors.

Being in a unique situation where we perform high-volume bariatric surgeries at a non-tertiary center, financial constraints have become a major limiting factor. Stapleless LSG seems a promising option to offset these constraints and to benefit the community. Based on the early experience of Shashank and colleagues, we would like to propose a stapleless laparoscopic sleeve gastrectomy technique.

A 36-Fr gastric bougie will be used for gastric calibration as per our standard sleeve gastrectomies. In this new approach, 1 to 1.5 cm distance from gastric bougie will be calculated for gastric transection. We have derived this measurement for suturing after calculating the possible lateral thermal spread which is 1 to 2 mm beyond the thermal device (LigaSure) and the seal width of 3 mm on either side of the blade. Therefore, the suturing of the first layer requires a minimum of 4–5 mm distance from the sealed/partially sealed gastric wall edge (a visual marker was also used—white coagulated tissue transition line/zone on anterior and posterior walls). The remaining 1 cm of tissue will be for imbricating the first layer. The above measurements also took into consideration the width of standard stapler devices which typically measures 1 cm in width and 5 mm on each side of the blade (often extra tissue is left to facilitate reinforcement of staples line). No guideline exists as to the amount of tissue needed so far.

Next, interrupted stay sutures are placed every 3 to 4 cm along the margin with 2/0 absorbable sutures, Polysorb (glycolic and lactic acid, Covidien) to keep anterior and posterior neogastric walls well oriented for suturing. For the first layer, continuous intermittent locking suturing is done from transected cardio-fundic area to antrum with 2/0 Polysorb sutures. For the second layer, extra mucosal running suturing is done, imbricating the first layer with 2/0 Unidirectional V-LOC, 30 cm (Covidien) suture. The V-LOC loop was not utilized; instead, knotting was done to ensure secure locking of suture end, thus, replicating standard open general surgical principles of tissue transection and suturing, laparoscopically.

We plan to conduct a pilot study on stapleless LSG to assess its safety and efficacy on patients. In view of the low cost needed to perform the surgery, we do hope that more patients can benefit from bariatric surgery which is known as the effective surgical method to reduce weight in obese patients and improve obesity-related co-morbidities.
